# Assessment of Betulinic Acid Cytotoxicity and Mitochondrial Metabolism Impairment in a Human Melanoma Cell Line

**DOI:** 10.3390/ijms22094870

**Published:** 2021-05-04

**Authors:** Dorina Coricovac, Cristina Adriana Dehelean, Iulia Pinzaru, Alexandra Mioc, Oana-Maria Aburel, Ioana Macasoi, George Andrei Draghici, Crina Petean, Codruta Soica, Madalina Boruga, Brigitha Vlaicu, Mirela Danina Muntean

**Affiliations:** 1 Faculty of Pharmacy, “Victor Babeș” University of Medicine and Pharmacy Timișoara, Eftimie Murgu Square No. 2, RO-300041 Timișoara, Romania; dorinacoricovac@umft.ro (D.C.); cadehelean@umft.ro (C.A.D.); macasoi.ioana@umft.ro (I.M.); draghici.george-andrei@umft.ro (G.A.D.); crina.petean@umft.ro (C.P.); codrutasoica@umft.ro (C.S.); 2 Research Center for Pharmaco-toxicological Evaluations, Faculty of Pharmacy, “Victor Babes” University of Medicine and Pharmacy Timișoara, Eftimie Murgu Square No. 2, RO-300041 Timișoara, Romania; 3 Faculty of Medicine “Victor Babeș” University of Medicine and Pharmacy Timișoara, Eftimie Murgu Square No. 2, RO-300041 Timișoara, Romania; oanaduicu@umft.ro (O.-M.A.); madalina.boruga@umft.ro (M.B.); vlaicu@umft.ro (B.V.); daninamuntean@umft.ro (M.D.M.); 4 Center for Translational Research and Systems Medicine, Faculty of Medicine,” Victor Babeș” University of Medicine and Pharmacy Timișoara, Eftimie Murgu Sq. no. 2, RO-300041 Timișoara, Romania

**Keywords:** betulinic acid, melanoma, mitochondria, OXPHOS, glycolysis, apoptotic markers, mitochondrial membrane potential

## Abstract

Melanoma represents one of the most aggressive and drug resistant skin cancers with poor prognosis in its advanced stages. Despite the increasing number of targeted therapies, novel approaches are needed to counteract both therapeutic resistance and the side effects of classic therapy. Betulinic acid (BA) is a bioactive phytocompound that has been reported to induce apoptosis in several types of cancers including melanomas; however, its effects on mitochondrial bioenergetics are less investigated. The present study performed in A375 human melanoma cells was aimed to characterize the effects of BA on mitochondrial bioenergetics and cellular behavior. BA demonstrated a dose-dependent inhibitory effect in both mitochondrial respiration and glycolysis in A375 melanoma cells and at sub-toxic concentrations (10 μM) induced mitochondrial dysfunction by eliciting a decrease in the mitochondrial membrane potential and changes in mitochondria morphology and localization. In addition, BA triggered a dose-dependent cytotoxic effect characterized by apoptotic features: morphological alterations (nuclear fragmentation, apoptotic bodies) and the upregulation of pro-apoptotic markers mRNA expression (Bax, Bad and Bak). BA represents a viable therapeutic option via a complex modulatory effect on mitochondrial metabolism that might be useful in advanced melanoma or as reliable strategy to counteract resistance to standard therapy.

## 1. Introduction

Cutaneous malignant melanoma, the most fatal form of skin cancer, is portrayed as a heterogeneous disorder based on distinct genomes, transcriptomes, proteomes, and epigenomes found within a tumor and, also, the presence of cellular plasticity, features that proved to be real-challenging for the novel therapeutic agents [1,2,3]. According to the European Cancer Information System (ECIS) rapport, in 2020, the estimated incidence of melanoma new cases was of approximative 210,000, the highest rates being reported for Northern European countries (Denmark and Sweden—50 to 42.1: 100,000 people) and Netherlands (48.5: 100,000), whereas the lowest numbers were accounted in the South-Eastern European countries (Bulgaria and Romania—9.0 and 7.9, respectively) [4]. A major concern regarding melanoma is represented by the advanced metastatic tumors that are unresectable and become very rapidly resistant to treatment (after several months only) due to the multiple mutations acquired that impact on proliferation, survival, and metastasis [2]. Despite the current available therapies and the progress recorded in melanoma treatment and diagnostic tools (targeted therapies for BRAF-mutated melanomas—vemurafenib, dabrafenib; immunotherapy—ipilimumab, nivolumab, pembrolizumab; and combined therapies), invasive melanoma remains the primary cause of skin cancer deaths and a continuous increment of melanoma incidence was recorded worldwide, even though the mortality rates decreased in the latest years [5]. All these facts suggest an urgent need for improvement of existing methods for melanoma prevention and protection, as well as to discover new diagnostic markers and innovative therapies that will bypass the resistance of melanoma cells and reduce the side effects of current therapies [6]. An innovative and feasible strategy for melanoma treatment consists in understanding the tumor cellular energetic metabolism, mainly the role of mitochondria in melanoma, a topic of great interest in recent years.

Mitochondria are versatile organelles that exert multiple functions within mammalian cells, as: (i) suppliers of cellular ATP (adenosine triphosphate) by oxidative phosphorylation, (ii) modulators of Ca^2+^ homeostasis, (iii) regulators of apoptosis via the intrinsic pathway, and iv) a source of reactive oxygen species (ROS) with both modulator and deleterious roles [7]. According to recent evidence, mitochondrial metabolism was upgraded from a simple bystander in the oncogenic process to a fundamental player in all the phases of the tumoral process including malignant transformation, tumor progression, and treatment efficiency [8]. The implication of mitochondria in melanoma development resides in the metabolic plasticity displayed by melanoma cells in the presence of different activators (AKT (protein kinase B), BRAF (v-raf murine sarcoma viral oncogene homolog B1), p14ARF, MYC, NRAS (neuroblastoma RAS viral oncogene homolog), phosphatidylinositol-4,5- bisphosphate 3 kinase catalytic subunit a (PIK3CA), and phosphatase and tensin homolog (PTEN)) [9], as follows: (i) BRAF-mutant melanomas exhibit a glycolytic phenotype that switches to oxidative phosphorylation (OXPHOS) in the presence of BRAF inhibitors; (ii) NRAS-mutant melanomas rely on aerobic glycolysis, and (iii) melanomas expressing peroxisome proliferator-activated receptor γ, coactivator 1α (PGC-1α) or PTEN present enhanced OXPHOS associated with a reduced glycolytic profile [6,10,11,12]. Moreover, melanoma cells proved to be highly adaptable in a metabolic manner, in response to different environmental conditions, as hypoxia or low extracellular glucose. Taken together, these findings underline the dynamic function of mitochondria in melanoma growth/progression/survival, treatment responses and resistance development [6,7,13] and the urge to get a more detailed picture of melanoma cells altered energy metabolism to overcome the drawbacks.

Agents that target mitochondria or interfere with mitochondria bioenergetics could be considered an alternative therapy that in combination with the current antimelanoma treatments augment the chemotherapeutics efficacy and delays the occurrence of resistance. A compound that complies with these requirements is betulinic acid (BA). The pentacyclic lupane-type triterpenoid, betulinic acid (3β-hydroxy-lup-20(29)-en-28-oic acid), exerts an extensive pharmacological profile that comprises: anticancer, anti-inflammatory, anti-bacterial, anti-HIV, anti-helminthic, and antiangiogenic effects. In addition, BA also exhibits anti-diabetic, anti-dyslipidemic, and other effects as summarized in three excellent reviews [14,15,16]. The features that make BA a very attractive compound for researchers in the field of carcinogenesis are the high toxicity for cancer cells and the very low toxicity on normal cells (as demonstrated in vitro on dermal fibroblasts and peripheral blood lymphocytes and in vivo on normal cells/organs at relatively high therapeutic doses, respectively) [17]. 

Studies performed over the past decade stated that BA inhibits the growth and induces apoptosis in multiple human cancer cell lines, several mechanisms being reported here: (i) the induction of apoptosis in a caspases activation dependent manner, correlated with an upregulation of the pro-apoptotic proteins of Bcl-2 (B-cell lymphoma 2) family, Bax and Bcl-xs [18]; (ii) the activation of p38 and SAP/JNK (pro-apoptotic MAPK subgroups), with the involvement of ROS upstream of MAPK response [19], and (iii) the induction of ROS generation [14]. Elucidation of the BA’s antimelanoma mechanisms of action is far from being complete. Although its mitochondrial-dependent proapoptotic effect has been described, information regarding the modulation of mitochondrial bioenergetics is rather scarce.

This present work is intended to comprehensively characterize the effects of BA on mitochondrial bioenergetics and cellular behavior in A375 human melanoma cells.

## 2. Results

### 2.1. High Concentrations of BA Promote Cytotoxicity and Morphological Changes in HaCaT Cells

**Figure 1:**
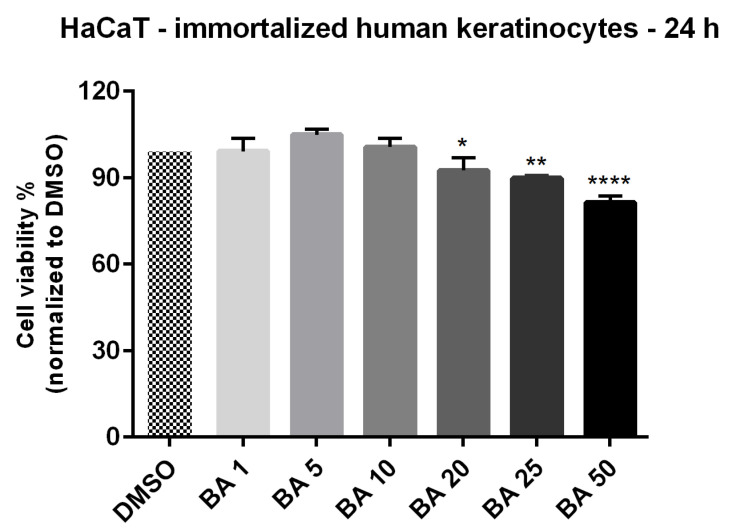
In vitro viability evaluation of BA (1, 5, 10, 20, 25 and 50 μM) in HaCaT cells at 24 h post-stimulation by MTT assay. The results are expressed as cell viability percentage (%) normalized to control (DMSO-stimulated) cells. The data represent the mean values ± SD of three independent experiments performed in triplicate. One-way ANOVA analysis was applied to determine the statistical differences in rapport with DMSO followed by Dunnett’s multiple comparisons post-test (* *p* < 0.05, ** *p* < 0.01 and **** *p* < 0.0001).

The findings of previous studies regarding the lack of toxicity induced by BA treatment in normal cells [20,21,22] are debatable. On this line, we verified the impact of BA treatment for 24 h on HaCaT (human immortalized) healthy cells’ viability by employing the MTT assay. Incubation of HaCaT cells with different concentrations of BA led to the following results: the lowest concentrations tested (1, 5 and 10 μM) did not affect cells viability, whereas higher doses (20, 25 and 50 μM) induced a dose-dependent decrease of cells viability, the percentage of viable cells calculated at the highest concentration tested—50 μM was 81.42% (see Figure 1). The results were normalized to DMSO, the solvent that was used to solubilize BA. 

**Figure 2:**
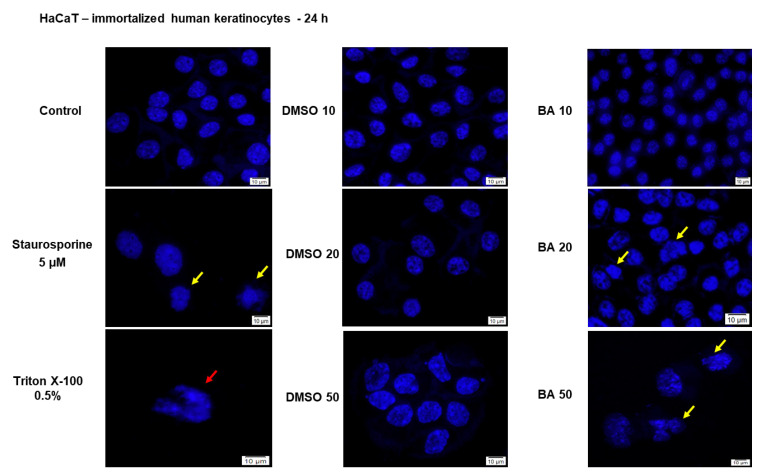
Cell nuclei staining using Hoechst 33342 in HaCaT cells after treatment with BA (10, 20 and 50 µM) and DMSO for 24 h. The pictures were taken at 24 h post-treatment. Staurosporine solution (5 µM) represents the positive control for apoptotic changes at nuclear level and Triton X-100 solution (0.5%) for necrosis. The yellow arrows represent signs of apoptosis as nuclear shrinkage or nuclear fragmentation, and the red arrow indicates signs of necrosis as cellular membrane disruption. The scale bar was 10 µm.

Another morphological hallmark for the cytotoxicity of a compound, is represented by the nuclear changes that indicate the presence of apoptotic or necrotic cells. To verify the type of cell death induced by BA (10, 20 and 50 μM—the concentrations were selected based on the cell viability results) in HaCaT cells, the nuclei were stained using Hoechst 33342 dye (Figure 2). As positive control for apoptosis induction was used Staurosporine solution (5 µM), and for necrosis—Triton X-100 solution (0.5%). Signs of apoptosis, as nuclear shrinkage or nuclear fragmentation (yellow arrows) were observed only in HaCaT cells treated with the highest concentrations of BA—20 and 50 µM, whereas BA 10 µM had no impact on HaCaT cells nuclei—the nuclei presented a round shape and no sign of chromatin condensation or blebbing, their aspect being similar with the one presented by the control cells and DMSO. No signs of necrosis (red arrow) were detected in HaCaT cells treated with BA or DMSO (Figure 2). Taken together, these results indicate that low concentrations of BA (1–10 µM) have no impact on HaCaT cells viability and morphology, whereas higher concentrations (20, 25, and 50 µM) reduce cells viability and induce morphological alterations (loss of contact with neighboring cells, cells shrinkage, nuclear fragmentation) specific for apoptotic death.

### 2.2. BA Exerts a Dose-Dependent Cytotoxic Effect in A375 Human Melanoma Cells

**Figure 3:**
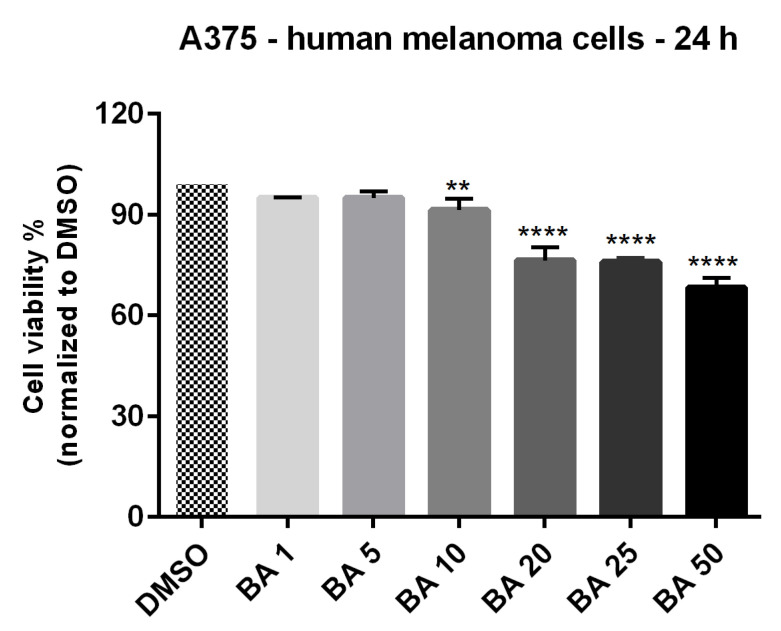
In vitro viability evaluation of BA (1, 5, 10, 20, 25 and 50 μM) in A375 cells at 24 h post-stimulation by MTT assay. The results are expressed as cell viability percentage (%) normalized to control (DMSO-stimulated) cells. The data represent the mean values ± SD of three independent experiments performed in triplicate. One-way ANOVA analysis was applied to determine the statistical differences in rapport with DMSO followed by Dunnett’s multiple comparisons post-test (** *p* < 0.01 and **** *p* < 0.0001).

To decipher the BA antimelanoma mechanism of action, we selected as experimental model the A375—human melanoma cell line. Compared to control group (DMSO-treated cells) BA treatment (24 h) determined a dose-dependent reduction in cell viability percentage (Figure 3). The decrease of cells’ viability percentage was observed starting at 10 μM (91.39%), but the lowest percentage of viable cells was recorded at the highest concentration tested—50 μM (68.22%). The calculated IC_50_ was 16.91 μM.

**Figure 4:**
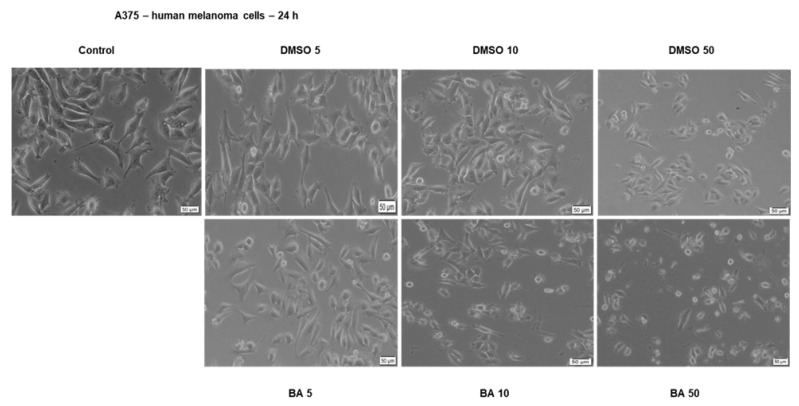
Representative images of the morphological aspect of A375 cells after treatment for 24 h with BA and DMSO (5, 10 and 50 μM). The scale bar was 50 μm.

Since BA treatment exerted a dose-dependent cytotoxic effect in A375 cells, we also verified its impact in terms of morphological alterations (Figure 4). The presence of several roundish and detached cells, but unmodified adherence and cell–cell contact was noticed at 10 μM BA concentration as compared to control cells (untreated cells). The highest BA concentration tested—50 μM induced significant morphological changes characterized by the presence of round cells floating, loss of cell–cell adhesions, loss of adherence, reduced confluence, and cellular debris (Figure 4), clear signs of cytotoxicity.

No morphological changes were detected in the DMSO-treated cells (solvent used for BA solubilization), their morphology and shape being similar to that of control cells (untreated): adherent and confluent cells, with an epithelial morphology (Figure 4).

The pictures presented in Figure 4 were selected as representative concentrations to clearly observe the changes that took place in melanoma cells morphology after BA treatment.

**Figure 5:**
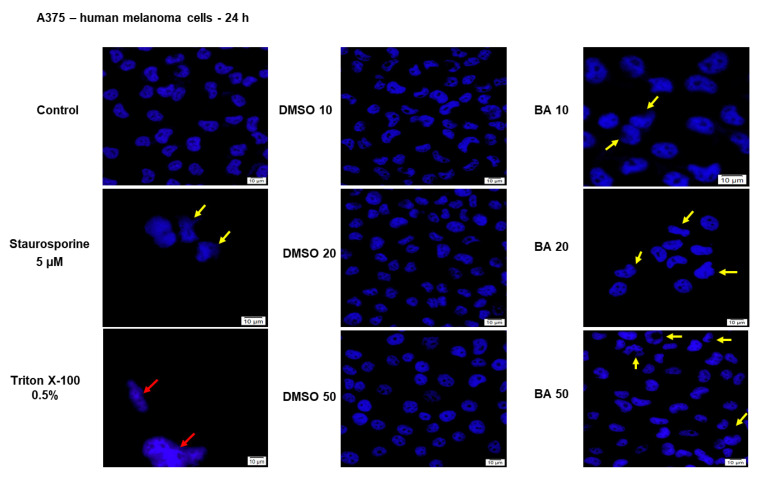
Nuclear staining using Hoechst 33342 in A375 cells after treatment with BA (10, 20 and 50 µM) and DMSO for 24 h. The pictures were taken at 24 h post-treatment. Staurosporine solution (5 µM) represents the positive control for apoptotic changes at nuclear level and Triton X-100 solution (0.5%) for necrosis. The yellow arrows represent signs of apoptosis as nuclear shrinkage or nuclear fragmentation, and the red arrow indicates signs of necrosis as cellular membrane disruption. The scale bar was 10 µm.

To clearly delineate the type of cell death triggered by BA treatment, we decided to perform nuclear staining using Hoechst 33342 dye (Figure 5).

The BA concentrations selected to perform this assay were 10, 20 and 50 μM based on cell viability and cytotoxicity results. Our results indicated that with increasing concentrations the apoptotic signs become more evident, as follows: nuclear shrinkage (a morphologic hallmark of apoptosis onset—BA 10 μM), nuclear fragmentation, presence of apoptotic bodies and chromatin condensation (yellow arrows), features similar with the ones observed after Staurosporine (5 μM) treatment—the positive control for apoptosis induction. No signs of necrosis (red arrows) were identified in BA-treated cells at the concentrations tested (Figure 5).

These results suggest that BA triggers a dose-dependent cytotoxic effect in melanoma cells by reducing cells viability and inducing apoptotic features: morphological alterations of both the nucleus and cytoplasm.

### 2.3. BA Treatment Impacts on Mitochondrial Membrane Potential in a Dose-Dependent Manner

**Figure 6:**
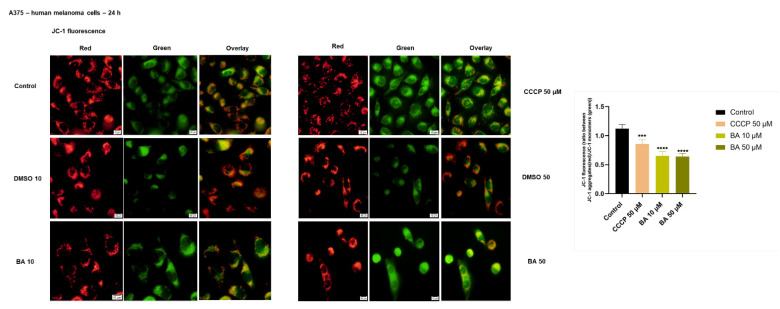
Mitochondrial membrane potential evaluation using JC-1 mitochondrial staining in A375 cells after treatment with BA (10 and 50 µM) and DMSO for 24 h. The pictures were taken at 24 h post-treatment. CCCP (50 µM) represents the positive control for mitochondrial depolarization. The graph represents the quantification of fluorescence intensity expressed as JC-1 aggregates (red)/JC-1 monomers (green) ratio. The data represent the mean values ± SD of four independent measurements. One-way ANOVA analysis was applied to determine the statistical differences in rapport with control group followed by Dunnett’s multiple comparisons post-test (*** *p* < 0.001 and **** *p* < 0.0001).

The apoptotic-like cytotoxicity induced by BA treatment in A375 cells and the previously described capacity of BA to induce apoptosis via the mitochondrial pathway, prompted us to check the impact of BA on the mitochondrial membrane potential integrity that represents a hallmark of apoptosis-related cell death. The JC-1 mitochondrial fluorescent probe was used to highlight the effects of BA. The predominance of red fluorescence indicates polarized mitochondria and cells unaffected by apoptosis, whereas the predominance of green fluorescence indicates depolarized mitochondria and dead cells. Our findings showed that BA-treated cells with 50 μM were predominantly green, what indicates a clear depolarization of mitochondria, a similar profile being also observed in 10 μM BA-treated cells but also the red fluorescence was detected (Figure 6). DMSO-treated cells at both concentrations tested (10 and 50 μM) exhibited a fluorescent aspect (predominantly red) as control cells (untreated), indicating viable cells with polarized mitochondria (Figure 6). The red (JC-1 aggregates)/green (JC-1 monomers) ratio was >1 in control cells what indicates healthy cells with polarized mitochondria, whereas in the other groups of cells (BA 10 and 50 µM, and CCCP—treated) the ratio was <1 (Figure 6).

### 2.4. BA Stimulation Induces Changes in Mitochondrial Markers mRNA Expression

**Figure 7:**
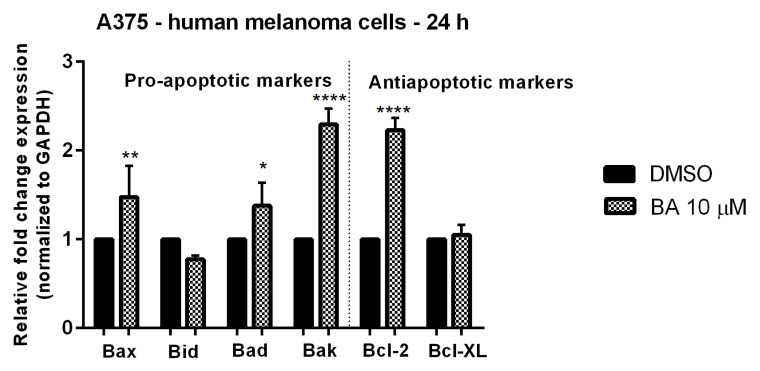
Relative fold expression of mRNA expression of pro- and anti-apoptotic mitochondrial markers in A375 cells after stimulation with BA (10 µM) for 24 h. The expressions were normalized to GAPDH and DMSO was used as control. Data represent the mean values ± SD of three independent experiments. One-way ANOVA with Dunnett’s post-test was applied to determine the statistical differences in rapport with DMSO stimulated cells (* *p* < 0.05, ** *p* < 0.01, and **** *p* < 0.0001).

Since it is known that BA induces apoptosis in cancer cells via the intrinsic pathway involving mitochondria and the data regarding the role played by the pro- and anti-apoptotic markers in this process is still debatable, we further verified the effect of BA on pro-apoptotic (Bid, Bax, Bak, and Bad) and anti-apoptotic markers (Bcl-2 and Bcl-XL) mRNA expression (Figure 7). The sub-cytotoxic concentration of BA—10 μM elicited an up-regulation of mRNA expression for most of the pro-apoptotic markers (Bax, Bad and Bak), except for Bid, the most significant increase in expression being calculated for Bak. BA treatment had no impact on Bcl-XL anti-apoptotic marker, whereas in the case of Bcl-2 it was also observed an upregulation (Figure 7). Moreover, we verified the impact of BA on caspase 3, caspase 8 and Apaf 1 mRNA expression and noticed a downregulation of these markers’ expressions in BA-treated cells (10 μM) as compared to DMSO-treated cells (data not shown) that did not reach statistical significance.

### 2.5. Impact of BA on Human Melanoma Cells Morphology and Cellular Organelles

**Figure 8:**
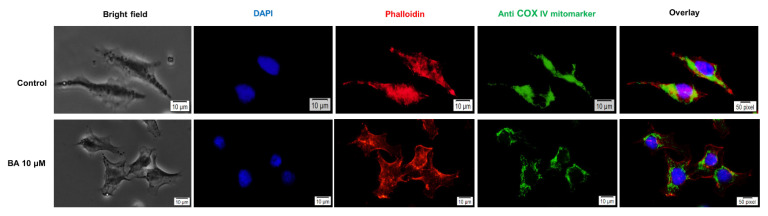
The impact of BA (10 µM) 24 h stimulation in A375 cells on: (a) nuclei—DAPI staining (blue), (b) F-actin fibers—Phalloidin (red) and (c) COX IV mitochondrial marker (green). The pictures were taken using 40× objective at a scale bar of 10 µm.

Changes in mitochondria morphology and localization are common features in melanoma cells as a consequence of the tumor metabolic reprogramming. To verify the localization of mitochondria in human melanoma cells—A375, was used a specific antibody—Anti COX IV mitomarker (green). In control cells, the fluorescence distribution was detected throughout the cell, whereas in the presence of BA it was concentrated around the nucleus, what could be considered that mitochondria are localized perinuclear (Figure 8). Phalloidin staining used to visualize F-actin fibers, indicated a slightly different distribution of these fibers in BA-stimulated cells as compared to control cells (unstimulated): the fluorescence is more concentrated at the edges of the cells as compared to control cells that present an evenly distribution of these fibers in the whole cell (Figure 8). The DMSO-treated presented a similar distribution of both mitochondria and actin fibers as the control cells (data not shown).

### 2.6. BA Treatment Modulates the Bioenergetic Profile of A375 Cells

**Figure 9:**
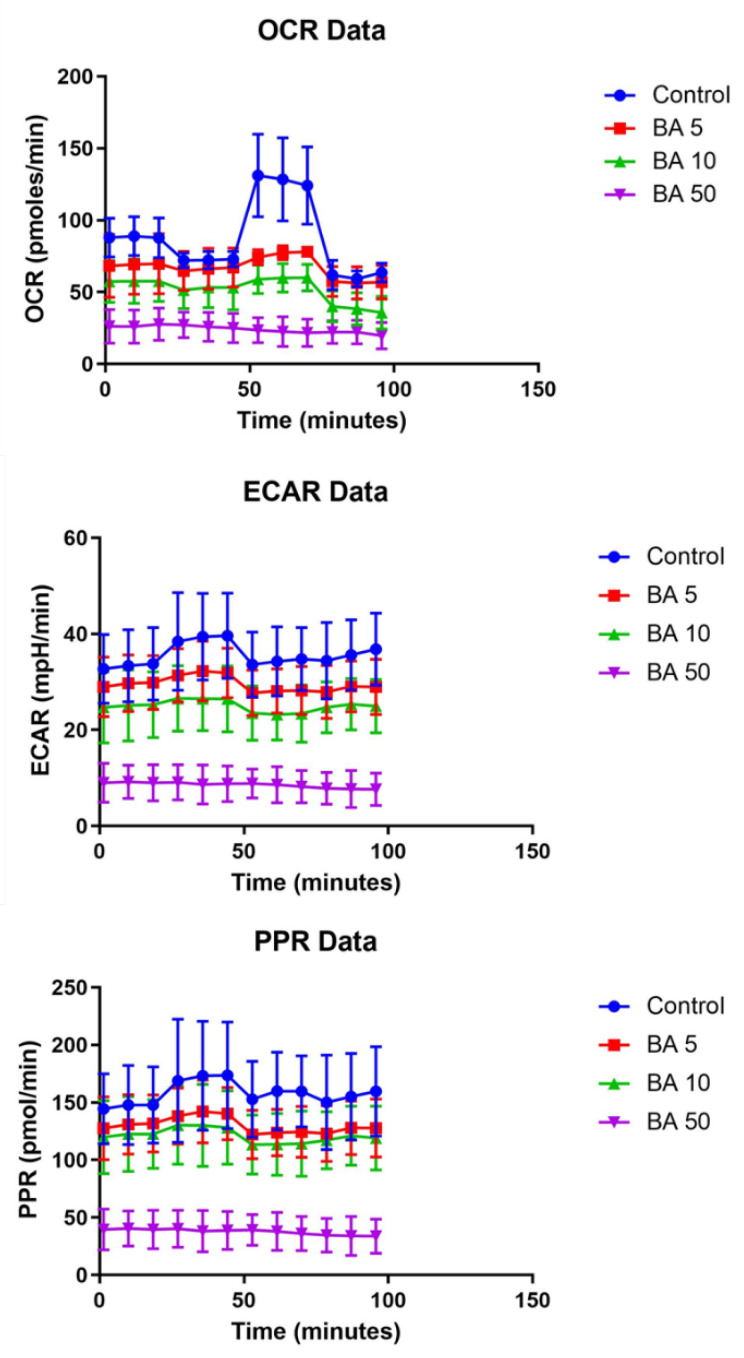
Summary traces of the bioenergetic profile of A375 cells: Control—untreated cells; BA 5—cells treated with 5 µM betulinic acid; BA 10—cells treated with 10 µM betulinic acid; BA 50—cells treated with 50 µM betulinic acid. OCR was expressed in units of nmoles/min/no of cells, ECAR in mpH/min/no of cells, and PPR in pmoles/min/no of cells.

In our study, the Seahorse XFe24 extracellular flux analyzer was used to evaluate the bioenergetic profile of A375 human melanoma cells treated for 24 h with BA in increasing concentrations (5, 10, and 50 µM). Both cellular OCR (oxygen consumption rate), the indicator of oxidative phosphorylation, and ECAR (extracellular acidification rate), the marker of glycolytic metabolism, were simultaneously monitored; the ECAR values were automatically converted to proton production rates (PPR) in order to directly reflect the efflux of lactic acid (Figure 9). By inhibiting mitochondrial complex V, oligomycin injection blocks the intracellular ATP synthesis via OXPHOS which is translated in a decrease of OCR (Figure 9); thereby, energy production is shifted to glycolysis and ECAR increases (Figure 9). The injection of the uncoupling agent, FCCP, significantly increases OCR (Figure 9) as more O_2_ is consumed to pump the excess protons back across the mitochondrial membrane, while ECAR remains increased due to cells’ attempt to maintain their energy balance by using glycolysis to generate ATP (Figure 9). Antimycin A, by inhibiting mitochondrial CIII stops the electron flow in the ETC which drastically reduces OCR (Figure 9), while ECAR is not significantly modified (Figure 9), since cells shifted to a glycolytic state, in order to maintain their energy balance. As seen in Figure 9, the PPR profile was similar to ECAR’s one.

**Figure 10:**
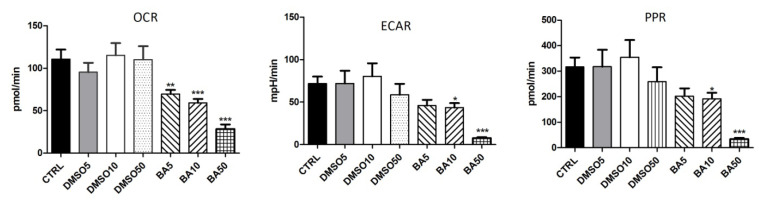
The effects of a 24 h treatment with BA (5, 10 and 50 µM) on OCR, ECAR, and PPR (n = 14–20/group. * *p* < 0.05 vs. untreated controls, ** *p* < 0.01, *** *p* < 0.001).

We showed here that both global OCR and ECAR (i.e., measured at the end of each experiment) were decreased in a concentration-dependent manner when A375 cells were treated with BA for 24 h, yet the concentration of 5 μM did not show a significant change of ECAR. Regarding the highest concentration of BA used in our experiments (50 μM), we found out that A375 cells had the lowest basal respiration and did not longer respond to the protocol used to modulate both glycolytic and mitochondrial pathways for ATP production (Figure 9); accordingly, the global OCR and ECAR presented the highest decline as compared to the untreated cells (Figure 10). In the end, the proton production rates (PPR) underwent changes very similar to ECAR in cells treated with BA. A limiting problem linked with BA is the poor water solubility. Thereby, BA was dissolved in DMSO, and our data showed that all tested DMSO concentrations did not interfere with the bioenergetic profile of A375 cells (Figure 10).

### 2.7. BA-Mediated Changes on Mitochondrial Respiratory Parameters in A375 Human Melanoma Cells 

**Figure 11:**
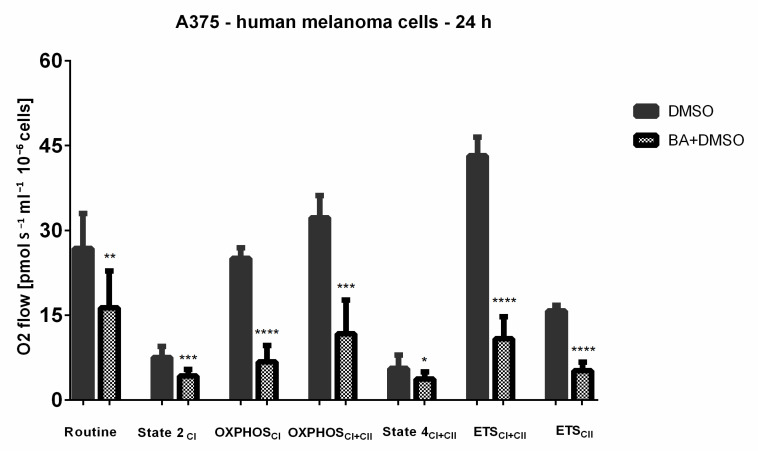
Respiration of permeabilized human melanoma cells—A375 following 24 h stimulation with BA (10 μM) and DMSO. Data represent the mean values ± SD of three independent experiments. Unpaired *t* test with Welch’s correction was applied to determine the statistical differences vs. DMSO-incubated cells (* *p* < 0.05, ** *p* < 0.01, *** *p* < 0.001, and **** *p* < 0.0001). The results were normalized to cells stimulated with DMSO (the vehicle used for BA solubilization). The following respiratory parameters were assessed: Routine—respiration of cells suspended in a substrate-free media; State 2_CI_—the respiration in basal conditions, OXPHOS_CI_—active respiration through CI; OXPHOS_CI+II_—maximal active respiration driven by CI and CII; State 4_CI+II_—basal respiration dependent on both CI and CII; ETS_CI+II_—maximal respiratory capacity of the electron transport system after FCCP titrations; ETS_CII_—electron transport system dependent only on CII.

To gain insights into the induced mitochondrial metabolic impairment in melanoma cells, we further evaluated the key parameters of mitochondrial respiration in permeabilized A375 cells treated with the optimal concentration of BA (10 μM) by the help of HRR (high-resolution respirometry). Our data showed that the stimulation of A375 cells with BA (10 μM) for 24 h determined a significant decrease of basal respiration rates—leak states (State 2_CI_ and State 4_CI+II_, measured after oligomycin addition) and routine respiration vs. DMSO-stimulated cells. A decline of active respiration (both OXPHOS_CI_ and OXPHOS_CI+II_) was also triggered by BA (Figure 11). Furthermore, our results indicate that BA significantly inhibited the maximal respiratory capacity (ETS_CI+II_) (Figure 11). Based on these findings, were calculated the corresponding respiratory flux ratios (R/E, L/E and RCR), indicators of mitochondrial coupling and OXPHOS efficiency, respectively (Table 1). The results summarized in Table 1 show that BA stimulation induced an increase of both R/E and L/E ratios, whereas RCR is decreased (Table 1).

Data represent the mean values ± SD of three independent experiments. Unpaired *t* test with Welch’s correction was applied to determine the statistical differences vs. DMSO-incubated cells (* *p* <0.05; ** *p* < 0.01).

## 3. Discussion

This study was particularly aimed at gathering novel insights regarding the effects of betulinic acid on mitochondrial bioenergetics in human melanoma cells, as a metabolism-driven approach to fully understand the antitumoral property of this bioactive phytocompound. The main working hypothesis was that the antimelanoma mechanism of action of BA involves the modulation of mitochondrial energetic metabolism. The major findings in this respect are as follows: (i) a dose-dependent inhibitory effect on both the oxygen consumption rate (OCR) and extracellular acidification rate (ECAR) (Figure 10) (the classic markers of oxidative phosphorylation and glycolysis, respectively) and (ii) a significant decrease elicited by a sub-toxic concentration (10 μM) of both complex I and II-supported respiratory parameters (in particular, the active respiration—OXPHOS, and the maximal respiratory capacity of the electron transfer system—ETS, respectively) (Figure 11 and Table 1). In addition, we demonstrated that BA elicited a dose-dependent cytotoxic effect characterized by apoptotic features: morphological alterations (nuclear fragmentation, apoptotic bodies—Figure 4 and Figure 5), a decrease in mitochondrial membrane potential (Figure 6), up-regulation of pro-apoptotic markers mRNA expression (Bax, Bad and Bak) (Figure 7) and changes in mitochondria morphology and localization in the A375 melanoma cell line (Figure 8).

Betulinic acid is included in the class of mitocans (abbreviation that was formed from mitochondria and cancer), agents that trigger cell death by targeting mitochondria [23]. BA has unequivocally proven its efficacy both in vitro and in vivo against a plethora of malignancies (melanoma, colorectal cancer, lung, hepatocellular, breast, prostate, stomach, pancreatic, neck and head carcinoma, ovarian, glioblastoma, chronic myeloid leukemia) and its selectivity, inducing no/very low effects on healthy/normal cells even at doses as high as 500 mg/kg body weight [17,24]. Moreover, BA formulated for topical application (20% betulinic acid ointment) was assessed in a phase I/II clinical trial for dysplastic nevi that can evolve towards melanoma; unfortunately, the study was suspended due to funding restriction [24,25,26]. 

View the key role played by metabolic pathways in cancer survival and progression, we assessed the mitochondrial effects of BA. In contrast to normal cells that rely on oxidative phosphorylation, tumor cells undergo a metabolic reprogramming, switching from oxidative phosphorylation to aerobic glycolysis [27]. In fact, two metabolic ‘programs’ have been reported to occur in tumors: one specific for actively proliferating cancer cells that require both oxidative phosphorylation and glycolysis (to facilitate the biosynthesis pathways that control proliferation) and a program specific for a subset of quiescent/slow-cycling cells that relies on mitochondrial respiration (to undergo continuous growth and gain intrinsic therapeutic resistance) [12]. It is the latter program that makes mitochondria a reliable target for the anticancer agents.

Indeed, it has been recently reported that both the aggressiveness of melanoma and resistance to treatment were assigned to the presence of a subset of cells that rely on respiration [12]. An augmented dependency on mitochondrial respiration was described in melanoma cells that express high levels of PGC1α (peroxisome proliferator-activated receptor γ coactivator 1α—a master regulator of mitochondria) and the BRAF gene [28,29]. By contrast, tumor cells that present a defective OXPHOS became extremely sensitive to cytotoxic drugs, since mitochondria assure from 40 to 75% of cancer cells ATP demands [12,27]. 

Targeted therapy that specifically inhibits BRAF and MEK pathways also inhibit glycolysis and is nowadays the mainstay of melanoma therapy; since an increase in oxidative phosphorylation has been reported to contribute to drug resistance, the inhibition of mitochondrial respiration has emerged as viable therapeutic strategy to overcome the resistance to pharmacological inhibitors [30,31]. Therefore, scientific efforts are aimed at discovery of compounds capable to inhibit both glycolytic and mitochondrial pathways for ATP generation [32,33]. 

In the present study, we analyzed the BA effects on human melanoma cells using the Seahorse XFe24 extracellular flux analyzer and showed the dose-dependent inhibition of both mitochondrial respiration and glycolysis. Our data are in line with a recent study demonstrating that citrate synthase activity, glycolysis rate and carbon flux through the TCA cycle were inhibited in BA-loaded nanoliposomes-treated colorectal cancer cells; the results were confirmed by the glycolysis stress test in which ECAR values revealed that both the basal glycolysis and the maximum glycolytic capacity were reduced by BA-loaded nanoliposomes [34].

In order to further dissect the effects of the phytocompound on mitochondrial respiration, we performed high-resolution respirometry studies (Oxygraph-2k, Oroboros Ltd., Innsbruck, Austria) using the standardized SUIT protocol in permeabilized human melanoma cells treated with 10 μM BA. To the best of our knowledge, this is the first study that demonstrates the inhibitory effect of BA on respiratory parameters in these cells. The above-mentioned concentration was selected based both on the Seahorse data and our previous publications where no toxicity was recorded for HaCaT cells (normal human keratinocytes).

As depicted in Figure 11, BA stimulation elicited an overall inhibition of cellular respiration in A375 melanoma cells by significantly decreasing the following parameters: basal respiration, active respiration, and maximal uncoupled respiration. The decrease of State 2 and State 4 (leak) suggests that the treatment with BA decreases oxygen consumption when the phosphorylation system is in an inactivated state, presumably due to a decrease in proton leak/slip across the mitochondrial membrane. Moreover, the significant decrease of both CI and CII-driven OXPHOS in parallel with the ETS mitigation unequivocally demonstrated that BA inhibits the active respiration and decreases the maximal respiratory capacity of the electron transfer system. A decline of maximal respiratory capacity is considered a reliable marker of a mitochondrial dysfunction [35]. Of note, the impairment of ATP production has been considered a promising therapeutic strategy for cancer since the compounds that act by inhibiting mitochondrial ATP production can trigger cell death in poorly perfused tumors and in those that highly rely on oxidative phosphorylation [36]. 

A similar mitochondrial dysfunction (inhibition of cellular respiration and impairment of mitochondrial electron transport chain complexes) in melanoma cells was described after stimulation with honokiol, a small molecule with anticancer properties, an activity that was associated with an augmented pro-apoptotic effect [37]. The impairment of oxidative phosphorylation and induction of oxidative stress was described as the mechanism exerted by resveratrol to inhibit the proliferation of different cancer cell lines (HeLa, MDA-MB-231, MCF7, SiHa and A549) [38]. Also, a recent paper showed that BRAF-mutant melanoma brain metastasis could be prevented by suppressing mitochondrial respiration [39].

Another druggable target in cancer is the phenomenon of uncoupling since increased uncoupling was related to tumor progression [40]. The uncoupling of oxidative phosphorylation is defined as significant reduction of the active respiration and a marked increase in the basal respiratory rates (with subsequent decrease in the respiratory control ratio). Interestingly, BA does not exert the effect of a typical uncoupler, described as an increased State 2 and State 4 respiratory rates [41,42,43], hence it induces mitochondrial dysfunction without increasing the proton transport across the inner mitochondrial membrane.

The flux respiratory ratios represent reliable indicators of changes that occur in mitochondrial respiration in response to different agents. The flux ratios that were calculated in the present study were: R/E—routine control ratio, L/E—leak control ratio, and RCR—respiratory control ratio. In normal cells, R/E ratio varies from 0.2 to 0.4 [44]. Values ≥ 0.5, as obtained for BA treated cells (0.975, Table 1), might suggest either an increased uncoupling, or defects in substrate oxidation/ETS complexes activity that led to the limitation of respiratory capacity, the latter being true for the case of BA. L/E ratio is an indicator of mitochondrial coupling with values as 0.0 indicating fully coupled mitochondria whereas 1.0 is attributed to fully uncoupled mitochondria [44]. The mean values of L/E obtained for BA treated cells (0.299, Table 1) suggest that human melanoma cells contain rather coupled mitochondria. As for the RCR ratio our results show a decrease in BA-treated A375 cells, demonstrating once more the mitochondrial dysfunction induced by BA. A decrease of mitochondrial membrane potential was induced by BA in a dose-dependent manner (Figure 6), finding that could be correlated with the reduction of maximal respiratory capacity following BA treatment.

The second important finding of the present study is represented by the BA-related toxicity in A375 human melanoma cells.

The cell viability results confirm the selective cytotoxicity of BA on human melanoma (68.22% viable A375 cells—IC_50_= 16.91 μM) and the low toxicity on normal cells (81.42% HaCaT viable cells) after a 24 h stimulation with the highest concentration of BA—50 μM (see Figure 1 and Figure 3). A cytotoxic effect of BA on melanoma cells was also described in other studies, with a range of IC_50_ between 1 to 76 μg/mL dependent on cell type [17,24]. Liebscher and coworkers proved that BA induced cytotoxicity not only on human melanoma cells—A375 (IC_50_ = 13.3 μM), but also on equine melanoma cell lines obtained from grey horses [45]. Our previous results showed that a concentration of 10 μM BA did not influence A375 cells viability but induced an epithelial-to-mesenchymal transition in these cells while a higher concentration (50 μM) proved to be toxic [46]. As regards the effect of BA on HaCaT cells, our data indicate that concentrations higher than 10 μM reduced cell viability and initiated morphological changes (cellular shape and nuclear alterations) (Figure 1). In a previous study conducted by Martins et al. [21] it was shown that BA (20 μM) triggers cell senescence in HaCaT cells by altering lipid bilayers, injuring mitochondria and lysosomes membranes and impairing autophagy. Taken together, these data support our cytotoxicity findings.

Since 1995, when BA was placed in the spotlight as an antimelanoma compound by Pisha et al. [47], a considerable number of studies were carried out to define the molecular pathways involved in BA-mediated antitumor activity, but several pieces are still missing from the complete picture. A mechanistic pathway that was initially suggested was induction of apoptosis via a direct effect on mitochondria involving Bcl-2 family, permeabilization of mitochondria outer membrane and activation of caspases [48]. The induction of apoptosis via the “intrinsic” or mitochondrial pathway is regulated by the Bcl-2 family of proteins. This family includes almost 20 members that are divided in anti-apoptotic (contain the four Bcl-2 homology domains—BH1-4: Bcl-2, Bcl-xL, Mcl-1—myeloid cell leukemia 1, A1 and Bcl-w) and pro-apoptotic (effector proteins with all BH1-4 domains: Bak and Bax and the BH3-only proteins: Bad, Bid, Bik, Bim, Bmf, bNIP3, HRK, Noxa and PUMA) proteins with regulatory functions in cell survival and apoptosis machinery by controlling the outer mitochondrial membrane permeabilization [49,50]. A deregulated function of Bcl-2 family is a common feature in different types of human cancers such as renal, stomach, and ovarian cancers, brain tumors, leukemia, and melanoma [16,51]. The anti-apoptotic members of Bcl-2 family act as keepers of the outer mitochondrial membrane integrity by linking and suppressing the activity of pro-apoptotic members, Bax (Bcl-2 associated x protein) and Bak (Bcl-2 antagonist killer 1) that promote the MOMP (mitochondrial outer membrane permeabilization) followed by apoptosis initiation [49].

Several lines of evidence suggest that BA modulates the Bcl-2 family proteins in a tumor-dependent manner, as follows: (i) upregulation of Bax pro-apoptotic protein in neuroblastoma, glioblastoma and melanoma cells; (ii) up-regulation of anti-apoptotic Bcl-2 protein in glioblastoma and neuroblastoma; (iii) no effect on Bak and Bad in melanoma cells and on Bcl-2 in squamous cell carcinoma cells; iv) increased expression of Mcl-1 protein in melanoma cells; (v) no significant effects on Bcl-xL in melanoma, neuroblastoma and glioblastoma cells; (vi) downregulation of Bcl-2 and Bcl-xL expressions in human multiple myeloma cells; and (vii) up-regulation of Bad expression and no effect on Bcl-xL in HeLa cells [52,53,54,55,56,57]. In the light of these findings, we assessed the effect of BA (10 µM) on the expression of both pro-apoptotic (Bax, Bak, Bad and Bid) and anti-apoptotic (Bcl-2 and Bcl-xL) markers. Accordingly, we showed that BA induced an up-regulation of Bax, Bad and Bak, and no effect on Bid expression in human melanoma cells. Regarding the anti-apoptotic marker Bcl-XL, its mRNA expression was not influenced by BA treatment, whereas in the case of Bcl-2 it was recorded an up-regulation (Figure 7). An interesting and unexpected finding was the upregulation of Bcl-2 expression by BA treatment. A similar case was described by Wick et al. in glioma cells [55]. An upregulation of another antiapoptotic marker, also a member of the Bcl-2 family, Mcl-1, by BA treatment was observed by Selzer et al. in melanoma cells [58], a phenomenon that could not be clearly explained by the authors, but they stated that BA-induced apoptosis was a late event that occurred after 24–48 h and the stimulation of Mcl-1 expression was observed in the first hours of treatment, and somehow speculated that this event could be an adaptative response of the cells to a cytotoxic stimulus.

It is well-known that a mitochondrial dysfunction is accompanied by phenotypical and morphological cellular modifications as an adaptive response to a potential injury [7] making the staining of actin microfilaments and tubulin fibers a mandatory test in the evaluation of compounds that target mitochondria. Actin microfilaments represent key components of the cytoskeleton that suffer conformational changes as a consequence of different cellular stressors and/or modifications of nucleotide binding [7]. To expand upon the findings stated before, we assessed the BA effect on actin microfilaments in A375 cells by performing immunofluorescence staining assays. Our results indicate a reorganization of actin microfilaments stained with phalloidin (red color) in BA-treated cells with a distribution focused mainly to the edges of the cells as compared to control and DMSO-treated cells that present a longitudinal uniform distribution within the cell (Figure 8). 

Mitochondria are dynamic organelles qualified to change the shape, size and subcellular localization in order to fulfill their particular biological and energetic demands [37].

The mitochondrial morphology in A375 cells was evaluated by the means of immunofluorescence staining in the presence of anti-COX IV antibody. MitoTracker (green) staining revealed in control and DMSO-treated cells a distribution of the mitochondria across the cellular cytoplasm, whereas in BA-treated cells, mitochondria appear tightly organized at the periphery of the nuclear envelope (Figure 8). These morphological changes were also described in other studies that investigated compounds targeting mitochondria such as ethidium bromide and honokiol [7,37]. Collectively, these findings confirm the impairment of mitochondrial morphology induced by BA stimulation in melanoma cells.

## 4. Materials and Methods

### 4.1. Reagents

The reagents applied in the present study: MgCl_2_×6H_2_O, EGTA (ethylene glycol-bis(β-aminoethyl ether)-N,N,N′,N′-tetraacetic acid), taurine, KH_2_PO_4_, lactobionic acid, sucrose, HEPES (4-(2-hydroxyethyl)-1-piperazineethanesulfonic acid), BSA (bovine serum albumin), digitonin, glutamate, malate, succinate, ADP (adenosine diphosphate), oligomycin, rotenone, antimycin A, FCCP (Carbonyl cyanide 4-(trifluoromethoxy)phenylhydrazone), betulinic acid (BA), and DMSO (dimethyl sulfoxide—the vehicle used for BA solubilization) were purchased from Sigma Aldrich, Merck KGaA(Darmstadt, Germany). All reagents used were of analytical purity. Cell culture media—Dulbecco’s modified Eagle Medium (DMEM) and specific supplements were acquired from Sigma Aldrich, Merck KGaA (Darmstadt, Germany), ATCC (American Type Cell Collection, Lomianki, Poland) and Thermo Fisher Scientific, Inc. (Waltham, MA, USA). XFe24 well plates, XF sensor cartridges, and XF Assay media were provided by Agilent Technologies, Inc. (Santa Clara, CA, USA).

### 4.2. Cell Lines and Cell Culture Conditions

The HaCaT—immortalized human keratinocytes and A375 (ATCC^®^ CRL-1619™)—human melanoma cell lines were purchased from CLS Cell Lines Service GmbH (Eppelheim, Germany) and American Type Culture Collection (ATCC, Lomianki, Poland), respectively. The cells were acquired as frozen items and kept in liquid nitrogen. HaCaT and A375 cells were cultured in specific media—Dulbecco’s modified Eagle Medium (DMEM) high glucose—4.5 g/L, supplemented with 10% fetal bovine serum (FBS) and antibiotic mixture (100 U/mL penicillin and 100 μg/mL streptomycin). The cells were kept in standard conditions: a humidified atmosphere with 5% CO_2_ at 37 °C. Cell number was determined automatically using the cell counting device, Countess II Automated Cell Counter (Thermo Fisher Scientific, Inc., Waltham, MA, USA), in the presence of Trypan blue.

### 4.3. Cytotoxicity Assessment

MTT assay. Cell viability was evaluated by using the MTT (3-(4,5-Dimethylthiazol-2-yl)-2,5-Diphenyltetrazolium Bromide) kit (Roche, Mannheim, Germany) according to the experimental protocol described in our previous studies [59]. In brief, HaCaT and A375 cells (1 × 10^4^/200 μL medium/well) were seeded in 96-well plates and stimulated with increasing concentrations (0–50 µM) of BA solubilized in DMSO for 24 h. The plates were incubated for 3 h at 37 °C with 10 µL/well of MTT reagent, followed by addition of buffer solution (100 µL/well) and incubation at room temperature and dark for 30 min. Absorbance measurement was performed at 570 nm using a xMark™ Microplate Spectrophotometer (Bio-Rad Laboratories, Inc., Hercules, CA, USA). The vehicle used for BA was dimethyl sulfoxide—DMSO for cell culture. The final concentration of DMSO did not exceed 1%. 

Evaluation of cellular morphology. Alterations of cellular morphology following the exposure to a test compound represent reliable clues for the identification of the type of induced cytotoxicity (i.e., cell death). Thus, we further monitored the impact of BA on cells’ morphology using the Olympus IX73 inverted microscope (Olympus, Tokyo, Japan). The images were taken at 24 h post-treatment and were analyzed using the cellSens Dimensions v.1.8. Software (Olympus, Tokyo, Japan) as described previously [60,61]. 

Hoechst nuclear staining. Apoptotic cells were labelled by the means of Hoechst 33342 nuclear staining assay (Invitrogen, Carlsbad, CA, USA). The protocol consisted of the following steps: (i) treatment of cultured cells—A375 and HaCaT (1 × 10^5^ cells/1.5 mL medium/well) with different concentrations of BA in DMSO (10, 20 and 50 µM) for 24 h; (ii) disposal of the medium containing the test compounds and addition of the staining solution—0.5 mL/well (1:2000 in PBS); (iii) a 10-min incubation at room temperature and dark, and (iv) the final washing step (3x) with PBS. The pictures were taken using Cytation 1 (BioTek Instruments Inc., Winooski, VT, USA) and the Olympus IX73 inverted microscope (Olympus, Tokyo, Japan). The images were analyzed using the Gen5™ Microplate Data Collection and Analysis Software (BioTek Instruments Inc., Winooski, VT, USA) and the cellSens Dimensions v.1.8. Software (Olympus, Tokyo, Japan). Staurosporine solution—5 µM—was used as positive control for apoptosis (3 h at 37 °C) and Triton X-100 (30 min at 37 °C)—0.5% for necrosis [62].

### 4.4. Mitochondrial Membrane Potential (ΔΨ) Assay (JC-1)

Mitochondrial membrane potential (ΔΨ) is considered a marker for cell health status and an essential indicator for mitochondrial function. To evaluate the impact of BA on ΔΨ in A375 human melanoma cells, we used the cationic JC-1 Dye (Mitochondrial Membrane Potential Probe, Invitrogen, Carlsbad, CA, USA). The assay was performed according to the experimental protocol described by Sivandzade et al. [63] and was adapted to our laboratory conditions. Briefly, the A375 cells were seeded in 12-well plates and were treated with different concentrations of BA and DMSO (10 and 50 µM) for 24 h. The JC-1 solution was added to reach a final concentration of 2 μM and the cells were incubated at 37 °C for 30 min. Visualization of the stained cells was performed using Olympus IX73 inverted microscope (Olympus, Tokyo, Japan). The red fluorescence mitochondrial signal was detected at 590 nm and is an indicator of JC-1 aggregates specific for polarized mitochondria (non-apoptotic cells). The green fluorescence is a specific marker for apoptotic and dead cells and was observed at an emission of 530 nm indicating that JC-1 dye remained in its monomeric form, a feature specific for depolarized mitochondria. The CCCP (carbonyl cyanide m-chlorophenylhydrazone) solution (50 μM, 5 min) was used as a positive control. The quantification of fluorescence intensity was calculated with ImageJ and Fiji software (National Institutes of Health, Bethesda, MD, USA) and the measurements of the images were performed by selecting a region of interest (ROI) that was measured by auto threshold according to the analysis software.

### 4.5. Quantification of Apoptotic Markers by Real-Time PCR

The total RNA content was extracted using peqGold RNAPureTM Package (Peqlab Biotechnology GmbH, Erlangen, Germany) according to the manufacturer’s instructions, and a DS-11 spectrophotometer (DeNovix, Wilmington, DE, USA) was used to measure the total concentration of RNA. Reverse transcription was performed using the Maxima^®^ First Strand cDNA Synthesis Kit (Thermo Fisher Scientific, Inc., Waltham, MA, USA) and the samples were incubated into Tadvanced Biometra Product line (Analytik Jena AG, Göttingen, Germany) according to the following thermal program: 10 min at 25 °C, 15 min at 50 °C and 5 min at 85 °C. The quantitative real-time PCR was conducted using a Quant Studio 5 real-time PCR system (Thermo Fisher Scientific, Inc., Waltham, MA, USA). The analysis was performed using 20 µL reactions containing Power SYBR-Green PCR Master Mix (Thermo Fisher Scientific, Inc., Waltham, MA, USA), samples’ cDNA, the sense and antisense primer and pure water. The following primer pairs were used (Table 2): 18S and GAPDH (as housekeeping genes), Bax, Bcl-2 (Thermo Fisher Scientific, Inc., Waltham, MA, USA), Bid, Bad, Bak, Bcl-xL, caspase 3, caspase 8 and Apaf-1 (Eurogentec, Seraing, Belgium). Normalized, relative expression data were calculated using the comparative threshold cycle (2-ΔΔCt) method.

### 4.6. Mitochondria Localization Using Immunofluorescence 

A375 cells (1 × 10^6^ cells/well) were seeded on a sterile slide in 6-well plates and stimulated with BA (10 µM) for 24 h. The staining protocol applied in the present study was based on a previous protocol [46] with several changes. Concisely, after the 24 h stimulation, the cells were washed three times with ice-cold PBS and fixed with paraformaldehyde 4% for 1 h at room temperature (RT). This step was followed by three times washing step with ice-cold PBS and permeabilization with a 2% Triton X/PBS solution for 30 min at RT. After washing with ice-cold PBS/0.01% Triton X, cells were blocked with 30% FBS in 0.01% Triton X for 1 h, followed by another washing step. To visualize the F-actin fibers, it was used Alexa Fluor^®^ 555 Phalloidin antibody (Cell Signaling Technology, Danvers, MA, USA) (incubation overnight at 4 °C using a 1:20 dilution). For the staining of COX IV—Mitochondrial marker it was used as primary antibody—Anti-COX IV antibody Mitochondrial marker (ab33985) (Abcam, Cambridge, United Kingdom) (incubation overnight at 4 °C using a 1:500 dilution), followed by the addition of the specific secondary antibody—Donkey Anti-Goat IgG H&L (Alexa Fluor^®^ 488—ab150129, Abcam, Cambridge, United Kingdom) next day for 2 h at room temperature and dark. The final step consisted of counterstaining the cells nuclei with 4′,6-diamidino-2-phenylindol (DAPI) for 15 min. 

### 4.7. Bioenergetic Profile of A375 Human Melanoma Cells 

A375 cells (1.5 × 10^4^ cells/well) were placed in XFe24 well culture plates and treated with increasing concentrations (5, 10, and 50 µM) of BA or DMSO (the BA vehicle), respectively; several wells were left untreated as control group. Background correction wells (i.e., wells that were not seeded with cells) were included in the assay to normalize the data to background plate noise. Plates were further placed in an incubator at 37 °C and 5% CO_2_ atmosphere, allowing the cells to adhere for 24 h. The analysis of the bioenergetic profile of A375 cells was performed in six in vitro experiments with the Seahorse XFe24 extracellular flux analyzer (Agilent Technologies, Inc., Santa Clara, CA, USA), an automatic platform that provides a simultaneous measurement of oxygen consumption rate (OCR) as the indicator of mitochondrial respiration and the extracellular acidification rate (ECAR) as the indirect measurement of anaerobic glycolysis according to a previously described method [64]. In brief, the metabolic activity of A375 cells was challenged with the classic modulators of the mitochondrial electron transport chain: the first automatic injection was performed with Oligomycin (1 µg/mL), the inhibitor of the mitochondrial ATP synthase; FCCP (3 µM), the classic uncoupler was further injected, followed by Antimycin A (5 µM), the inhibitor of mitochondrial complex III. OCR was reported in units of pmol min^−1^, while ECAR as mpH min^−1^ was automatically converted to proton production rate (PPR) expressed as pmol H^+^ min^−1^ using the determined buffer capacity of the media and the chamber volume in XFe24 Analyzer.

### 4.8. High-Resolution Respirometry Studies in Permeabilized Cells

Mitochondrial respiration was assessed in real time at 37 °C by means of high-resolution respirometry (HRR) (Oxygraph-2k; Oroboros Instruments GmbH, Innsbruck, Austria). Mitochondrial respiratory rates were obtained by following a SUIT protocol, adjusted to obtain both separate and conjunctive electron flow from complex I (CI) or/and complex II (CII). Prior to mitochondrial respiratory function assessment, the cells grown in T75 culture flasks were washed with PBS, trypsinized and resuspended in respiration medium (3 mM MgCl_2_ × 6H_2_O, 0.5 mM EGTA, 20 mM taurine, 10 mM KH_2_PO_4_, 60 mM lactobionic acid, 110 mM sucrose, 20 mM HEPES, 1 g/L BSA, pH 7.1, 37 °C) at a cell density (1 × 10^6^/mL) that yielded a routine respiration of at least 20 pmol/s per cm^3^ volume-specific oxygen flux for the untreated (control) cells. A stock solution of BA (5 mM) in DMSO was prepared and used to yield a final constant concentration of BA of 10 μM in the 2 mL chamber of the device. The concentration tested for DMSO was also 10 μM and did not exceed 0.2%. The titration of the substrates, inhibitors, and uncouplers was realized using Hamilton syringes (Hamilton Company, Reno, NV, USA). 

In order to study the extended functional OXPHOS, by allowing the passage of externally added compounds into cytosol, cell membrane was permeabilized with digitonin (35 μg/10^6^ cells). The optimal concentration used for a complete plasma membrane permeabilization was determined after a stepwise digitonin titration (data not shown), according to a protocol previously described by Pesta and Gnaiger [44]. The first respiratory rate recorded was routine respiration, obtained after the cells were suspended in a substrate-free media, for 10–15 min. The SUIT protocol applied consisted of the following steps: (1) plasma membrane permeabilization with digitonin; (2) addition of glutamate (G: 10 mM) and malate (M: 5 mM), as mitochondrial CI substrates; (3) addition of ADP (5 mM)—active respiration dependent on CI; (4) addition of succinate (S: 10 mM), a CII substrate—induced the maximal OXPHOS capacity, driven by both CI and CII; (5) inhibition of ATP synthesis induced by oligomycin (1 μg/mL), an ATP synthase inhibitor; (6) successive titrations of FCCP (1 μM/step), a classical uncoupling agent—the maximal respiratory capacity of the electron transport system (ETS); (7) addition of rotenone (0.5 μM), a CI inhibitor, permitted the measurement of electron transport system dependent only on CII, and (8) addition of a complex CIII inhibitor, antimycin A (2.5 μM), to completely inhibit the electron transport chain and to measure the residual oxygen consumption (ROX). All the respiratory rates obtained were corrected for ROX. Data were recorded using the DatLab software (Oroboros Instruments GmbH, Innsbruck, Austria). For a better understanding and interpretation of mitochondrial respiration, the following parameters were analyzed:

1. Routine respiration (basal respiration)—R—represents the physiological respiratory activity in intact cells; is dependent of cellular energy need, energy turnover and the degree of coupling to phosphorylation to ADP [65]
2. State 2_CI_—Leak state—L—basal respiration measured in the presence of malate (M) and glutamate (G)—complex I dependent—substrates (NADH-generating substrates).
3. OXPHOS_CI_ (active respiration)—P—mitochondrial respiratory capacity in the ADP-activated state of oxidative phosphorylation, measured after addition of ADP and in the presence of reducing complex I-dependent substrates: M and G.
4. OXPHOS_CII_ (active respiration)—mitochondrial convergent respiration through complex I and II; addition of ADP mentioned above was followed by addition of succinate (S), a complex II-dependent substrate [65,66].
5. State 4_CI+CII_—Leak state—obtained after addition of oligomycin—an inhibitor of ATP synthase, this residual respiration rate is assigned to proton leak, yields a non-phosphorylating state and a return to basal respiration dependent on both CI and CII.
6. ETS_CI+CII_—the maximal respiratory capacity of the electron transport system mimics a physiological energy need inducing an increase of oxygen consumption. It is acquired after successive titrations of an uncoupler as FCCP (carbonyl cyanide p-(trifluoro-methoxy) phenyl-hydrazone [67].
7. Residual oxygen consumption—ROX—indicates the remaining mitochondrial respiration after addition of rotenone—an inhibitor of complex I and antimycin—an inhibitor of complex III [67].
8. R/E (Routine/ ETS_CI+II_)—routine respiration normalized to ETS capacity; indicates how tight functions Routine respiration to the maximum capacity of the system.
9. L/E (State 4_CI+II_/ ETS_CI+II_)—Leak respiration normalized to ETS capacity; represents the part of maximum respiratory capacity linked to proton leak. Values as 0.0 indicate fully coupled mitochondria, whereas 1.0 is attributed to fully uncoupled mitochondria [44].
10. RCR—(OXPHOS_CI+II_/State 4_CI+II_)—respiratory control ratio is defined as the ratio between active respiration and leak state obtained after oligomycin addition; is strongly affected by any change in oxidative phosphorylation and is considered a useful indicator of mitochondrial dysfunction [35].

### 4.9. Statistical Analysis

The results obtained were expressed as means ± SD, and the difference between means was compared by one-way ANOVA, followed by Dunnett’s multiple comparison post hoc test and unpaired *t* test with Welch’s correction (GraphPad Prism version 6.0.0 for Windows, GraphPad Software, San Diego, CA, USA, www.graphpad.com, accessed on 18 March 2021). The difference between groups was considered statistically significant if *p* < 0.05 and are marked with * (* *p* < 0.05, ** *p* < 0.01, *** *p* < 0.001 and **** *p* < 0.0001). Data were expressed as means ± SEM. Data analysis used one-way ANOVA followed by a post-hoc Dunnett’s multiple comparison test (GraphPad Prism version 5.0.0 for Windows, GraphPad Software, San Diego, CA, USA, www.graphpad.com, accessed on 18 March 2021). The difference was considered statistically significant if *p* < 0.05.

## 5. Conclusions

Our results indicate that betulinic acid elicited a dose-dependent inhibitory effect on both mitochondrial respiration and glycolysis in A375 human melanoma cells. Mitochondrial bioenergetic dysfunction was associated with cytoskeleton reorganization (actin fibers), changes in mitochondrial morphology, a decrease of mitochondrial membrane potential and the upregulation of pro-apoptotic markers (Bax, Bad, and Bax). Taken together, these findings suggest that the molecular targeting of mitochondria bioenergetics with betulinic acid might represent a valid strategy for advanced melanoma and offer a novel perspective in understating the BA’s antimelanoma mechanism of action.

## Figures and Tables

**Table 1:** Flux control ratios calculated for permeabilized A375 cells treated with DMSO and BA—10 μM.

Flux Control Ratios	24 h Stimulation	
	DMSO	BA
R/E	0.604 ± 0.145	0.975 ± 0.022 **
L/E	0.154 ± 0.040	0.299 ± 0.098 *
RCR	4.530 ± 1.398	2.785 ± 0.953 *

CI—complex I; CII—complex II; R/E—Routine/ ETS_CI+II_; L/E—LEAK (State 4 _CI+II_)/ETS_CI+II_; RCR—OXPHOS_CI+II_ /LEAK (State 4 _CI+II_).

**Table 2:** The oligonucleotides of the primers used in the study.

Primer’s Name	Forward	Reverse
*18 S* *	5′ GTAACCCGTTGAACCCCATT 3′	5′ CCA-TCC-AAT-CGG-TAGTAG-CG 3′
*GAPDH* *	5′AAG-GTG-AAG-GTC-GGA-GTC-AAC 3′	5′GGG-GTC-ATT-GAT-GGC-AAC-AAT-A 3′
*Bax*	5′ GCCGGGTTGTCGCCCTTTT 3′	5′CCGCTCCCGGAGGAAGTCCA 3′
*Bid*	5′CCT-TGC-TCC-GTG-ATG-TCT-TTC 3′	5′GTA-GGT-GCC-TAG-GTT-CTG-GT 3′
*Bad*	5′CCC-AGA-GTT-TGA-GCC-GAG-TG 3′	5′CCC-ATC-CCT-TCG-TCC-T 3′
*Bak*	5′ATGGTCACCTTACCTCTGCAA 3′	5′TCATAGCGTCGGTTGATGTCG 3′
*Bcl-2*	5′CGGGAGATGTCGCCCCTGGT 3′	5′GCATGCTGGGGCCGTACAGT 3′
*Bcl-XL*	5′GATCCCCATGGCAGCAGTAAAGCAAG 3′	5′ CCCCATCCCGGAAGAGTTCATTCACT 3′
*Caspase 3*	5′GCGGTTGTAGAAGAGTTTCGTG 3′	5′CTCACGGCCTGGGATTTCAA 3′
*Caspase 8*	5′AGAGTCTGTGCCCAAATCAAC 3′	5′GCTGCTTCTCTCTTTGCTGAA 3′
*Apaf-1*	5′CACGTTCAAAGGTGGCTGAT 3′	5′TGGTCAACTGCAAGGACCAT 3′

* housekeeping gene.
